# Baseline Serum Cholesterol Is Associated with a Response to Pegylated Interferon Alfa-2b and Ribavirin Therapy for Chronic Hepatitis C Genotype 2

**DOI:** 10.1155/2012/317580

**Published:** 2012-11-05

**Authors:** Naota Taura, Tatsuki Ichikawa, Hisamitsu Miyaaki, Yoshiko Kadokawa, Takuya Tsutsumi, Shotaro Tsuruta, Yuji Kato, Osami Inoue, Noboru Kinoshita, Kazuo Ohba, Hiroyuki Kato, Kazuyuki Ohata, Junichi Masuda, Keisuke Hamasaki, Hiroshi Yatsuhashi, Kazuhiko Nakao

**Affiliations:** ^1^Department of Gastroenterology and Hepatology, Graduate School of Biomedical Sciences, Nagasaki University, Sakamoto 1-7-1, Nagasaki 852-8501, Japan; ^2^Department of Gastroenterology and Hepatology, Sasebo City General Hospital, Hirase-machi 9-3, Sasebo 857-8511, Japan; ^3^Department of Gastroenterology and Hepatology, Nagasaki Municipal Hospital, Shinchi-machi 6-39, Nagasaki 850-8555, Japan; ^4^Department of Gastroenterology and Hepatology, Japanese Red Cross Nagasaki Genbaku Hospital, Mori-machi 3-15, Nagasaki 852-8511, Japan; ^5^Department of Gastroenterology and Hepatology, Oita Prefectural Hospital, Bunyo 467, Oita 870-8511, Japan; ^6^Digestive Organ Center, Japan Labour and Welfare Organization, Nagasaki Labour Welfare Hospital, Setogoe 2-12-5, Sasebo 857-0134, Japan; ^7^Department of Gastroenterology and Hepatology, Sasebo Chuo Hospital, Yamato-machi 15, Sasebo 857-1195, Japan; ^8^Department of Internal Medicine, Goto Central Hospital, Nagasaki Prefectural, Yoshikugichou 205, Goto 853-0031, Japan; ^9^Department of Internal Medicine, National Hospital Organization Saga National Hospital, Hinode 1-20-1, Saga 849-8577, Japan; ^10^Department of Internal Medicine, Kouseikai Hospital, Hayama1-3-12, Nagasaki 852-8053, Japan; ^11^Department of Gastroenterology and Hepatology, Medical Inc. Kosei-kai Nijigaoka Hospital, Nijigaoka-machi 1-1, Nagasaki 852-8055, Japan; ^12^Department of Internal Medicine, Caritas Clinic, Nishiizuru-machi 65-7, Nagasaki 851-2322, Japan; ^13^Clinical Research Center, National Hospital Organization Nagasaki Medical Center, Kubara 2-1001-1, Omura, Nagasaki 856-8562, Japan

## Abstract

*Background*. HCV infection is associated with lipid disorders because this virus utilizes the host lipid metabolism to sustain its life cycle. Several studies have indicated that higher concentrations of serum cholesterol and LDL before treatment are important predictors of higher rates of sustained virological response (SVR). However, most of these studies involved patients infected with HCV genotype 1. Thus, we performed a multi-institutional clinical study to evaluate the impact of lipid profiles on SVR rates in patients with HCV genotype 2. *Methods*. A total of 100 chronic hepatitis C patients with HCV genotype 2 who received peg-IFN alfa-2b and ribavirin therapy were consecutively enrolled. The significance of age, sex, BMI, AST level, ALT level, WBC, hemoglobin, platelet count, gamma-glutamyltransferase, total cholesterol level (TC), LDL level, HCV RNA, and histological evaluation was examined for SVR using logistic regression analysis. *Results*. The 100 patients infected with HCV genotype 2 were divided into 2 groups, an SVR group and a non-SVR group. Characteristics of each group were subsequently compared. There was no significant difference in the level of HCV RNA, BMI, platelet, TG, or stage of fibrosis between the groups. However, there were significant differences in the levels of TC and LDL-C. In multivariate logistic regression analysis using baseline characteristics, high TC level was an independent and significant risk factor (relative risk 18.59, *P* = 0.015) for SVR. *Conclusion*. Baseline serum total cholesterol levels should be considered when assessing the likelihood of sustained treatment response following the course of peg-IFN and ribavirin therapy in patients with chronic HCV genotype 2 infection.

## 1. Introduction

Hepatitis C virus (HCV) causes acute and chronic hepatitis as well as liver cirrhosis and hepatocellular carcinoma [1]. A single-stranded RNA genome encodes 1 large open reading frame that is processed into at least 10 proteins by host and viral enzymes [2]. Some viral proteins are known to affect the outcome of pegylated interferon (PEG-IFN) and ribavirin combination therapy, which is the current standard for treating chronic hepatitis [3, 4].

HCV infection is associated with lipid disorders because this virus utilizes the host lipid metabolism to sustain its life cycle [5, 6]. Accordingly, understanding lipid metabolism in HCV infection is necessary for developing new strategies for complete eradication of this virus. Characteristic lipid disorders observed in chronic hepatitis C patients include steatosis and hypocholesterolemia, which are primarily caused by abnormal triglyceride (TG) and cholesterol metabolism, respectively [7]. The metabolic pathways of these 2 lipids are closely related to each other.

Several studies have indicated that higher concentrations of serum cholesterol and LDL before treatment are important predictors of high rates of sustained virological response (SVR) [8–10]. However, most of these studies involved patients who were infected with HCV genotype 1. Prognostic factors are likely to differ considerably between genotypes 1 and 2. For example, two studies have shown that total PEG-IFN and ribavirin doses are independent predictive factors of an SVR to the HCV genotype 1, whereas another found that dosages of PEG-IFN and ribavirin on SVR are not related to the genotype 2 [11, 12]. Total dosages of PEG-IFN and ribavirin may similarly influence the SVR to genotypes 1 and 2. Identifying factors involved in the responses of patients infected with HCV genotype 2 to PEG-IFN and ribavirin is important when considering treatment strategies. Fewer patients are infected with HCV genotype 2 than genotype 1. Thus, we performed a multi-institutional clinical study to evaluate the impact of lipid profiles on SVR rates in patients with HCV genotype 2.

## 2. Patients and Methods

### 2.1. Patients

A total of 685 patients with chronic hepatitis C diagnosed between 2004 and 2008 in the Nagasaki Association for the Study of Liver Disease (NASLD) were recruited for this study. All patients were included if they were positive for HCV antibodies and serum HCV RNA. One hundred patients with HCV genotype 2 who received pegylated interferon alfa-2b (PEG-INF) and ribavirin therapy were consecutively enrolled. Exclusion criteria were as follows: (1) positive for serum hepatitis B virus surface antigen, (2) abnormal thyroid and kidney functions, (3) decompensated liver disease, (4) presence of human immunodeficiency virus type I infection, and (5) ever received specific antiviral therapy prior to referral.

### 2.2. Study Protocol

This study is retrospective study. Response to antiviral treatment was assessed in patients based on HCV viremia and aminotransferase levels. Patients treated with a combination of PEG-IFN alfa-2b (product by MSD) and ribavirin received 1.0–1.5 *μ*g/kg and 600–800 mg daily of each drug, respectively. SVR was defined as both normal aminotransferase levels and undetectable serum HCV RNA 24 weeks after the end of antiviral therapy. The remaining patients were considered nonvirus responders (non-SVR).

Fasting serum samples were obtained in the early morning for biochemical analysis. Body mass index (BMI) was calculated as body weight in kilograms divided by the square of the height in meters (kg/m^2^). Liver biopsy specimens were fixed in 10% formalin, embedded in paraffin, cut to a thickness of 4 *μ*m, and stained with hematoxylin-eosin and Azan. All liver tissue specimens were evaluated by one pathologist who was unaware of patient clinical conditions. Liver histology was evaluated according to the degree of fibrosis and necroinflammatory activity [13]. The extent of fibrosis (staging) was classified as follows: F1 (periportal expansion), F2 (portoportal septa), F3 (portocentral linkage or bridging fibrosis), and F4 (cirrhosis). Necroinflammatory activity (grading) was classified as follows: A1 (mild), A2 (moderate), and A3 (severe). In order to define the cutoff parameter for total cholesterol level (TC), LDL, and TG for the SVR of PEG-IFN alfa-2b and ribavirin in HCV patients, we used the ROC curve. The area under the curve was 62% (CI 95%: 51%–75%), 72% (CI 95%: 59%–86%), and 61% (CI 95%: 46%–76%), respectively. The ideal cutoff point for the TC, LDL, and TG was calculated to be 177 with sensitivity equal to 58% and specificity equal to 77%, 98 with sensitivity equal to 57% and specificity equal to 77%, and 88 with sensitivity equal to 56% and specificity equal to 67%, respectively.

The protocol was approved by the Ethical Committee of the Nagasaki University School of Medicine.

### 2.3. Statistical Analysis

Descriptive summaries of study groups are reported as the median (range) and number (%). Data were analyzed using the Mann-Whitney *U* test for continuous ordinal data, and the chi-square test with Yates' correction and Fisher's exact test were performed for intergroup comparisons to determine the association between 2 qualitative variables. *P*-values <0.05 were considered statistically significant. Variables achieving statistical significance according to univariate analysis were subsequently included in the multivariate analysis using a logistic regression model and were described as relative risk (RR) with 95% confidence intervals (CI). Coefficients were calculated from the linear discriminating function of the variables. Data analysis was performed using SPSS version 16.0 for Windows.

## 3. Results

### 3.1. Patient Clinical Features

Baseline characteristics of the 100 patients infected with HCV genotype 2 are shown in Table 1. There were 54 male (54%) and 46 female (46%) patients, with a median age of 57 years.

The 100 patients infected with HCV genotype 2 were then divided into 2 groups, an SVR group (74 patients) and Non-SVR group (26 patients). Characteristics of each group were subsequently compared (Table 2). There was no significant difference in the level of HCV RNA, BMI, platelet, TG, or stage of fibrosis between the groups. However, there were significant differences in the level of TC and LDL-C.

### 3.2. Univariate and Multivariate Analysis of Factors Associated with SVR to Pegylated Interferon Alfa-2b and Ribavirin Therapy

Univariate and multivariate analysis in 100 patients infected with HCV genotype 2 was performed to identify independent factors relevant to an SVR (Table 3). In univariate analysis, the following 2 factors significantly influenced the SVR: TC (≥177 mg/dL; relative risk, 3.77; 95% confidence interval (95% CI), 1.41–10.05; *P* = 0.008) and LDL-C (≥98 mg/dL; relative risk, 4.91; 95% CI, 1.19–20.23; *P* = 0.028). However, in multivariate analysis, TC was the only independent factor for SVR (relative risk, 18.59; 95% CI, 1.78–193.65; *P* = 0.015).

### 3.3. Association of SVR Rate to Combination Therapy and TC Level

The 100 patients infected with HCV genotype 2 were then divided into 2 groups, a high serum TC level group (≥177 mg/dL) and a low serum TC level group (<177 mg/dL). Characteristics of each group were subsequently compared (Table 4). There was no significant difference in age, the level of ALT, WBC, hemoglobin, platelet, TG, stage of fibrosis or grade of inflammation between the groups. However, there were significant differences in sex, BMI, the level of AST, TG, LDL-C, and HCV RNA.

**Figure 1:**
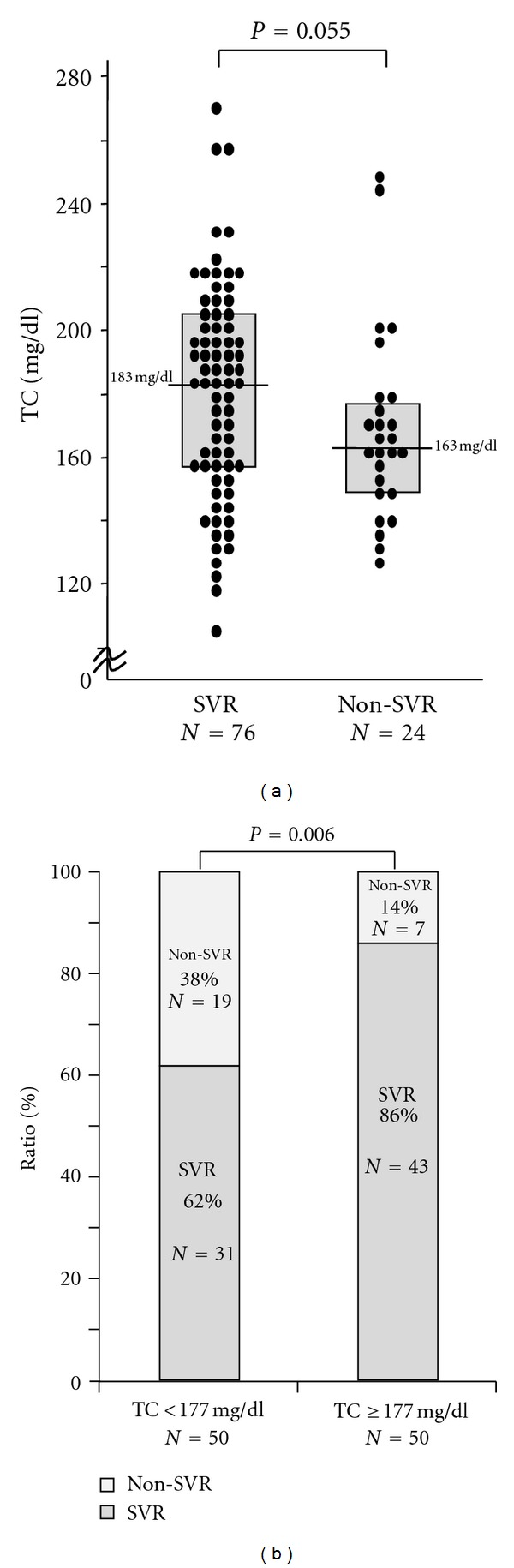
Comparison between SVR rate in patients with high serum TC levels (≥177 mg/dL) and patients with low serum TC levels (<177 mg/dL) in HCV genotype 2 patients.

We examined the differences in the 4 indices related to SVR rate between high serum TC level and low serum TC level in HCV genotype 2 patients (Figure 1). The SVR rate in low serum TC level patients was 62% (31 of 50), whereas 86% of patients (43 of 50) had serum high TC levels. The significantly higher SVR rate of serum high TC level than low serum TC levels was observed in 100 patients infected with HCV genotype 2.

## 4. Discussion

In this retrospective study, we showed a significant association of treatment response with baseline characteristics of patients infected with HCV genotype 2, including HCV viral load, BMI, and serum cholesterol level. Several baseline predictors for SVR have been identified in earlier studies [14–17]. Notably, among pretreatment features in the present study, serum TC levels appeared to discriminate responders from nonresponders independently of different treatment schedules. The response rate to standard treatment for patients with HCV genotype 2 using a combination of PEG-IFN and ribavirin is approximately 80% and remains a major concern in patient care. Our findings confirm serum high TC level as a good predictor of SVR in genotype 2. In patients with genotype 2, the SVR rate in patients with low serum TC levels was 62%, whereas 86% had high serum TC levels. Serum cholesterol as a predictor of SVR in patients with chronic hepatitis C is in accordance with the results of previous studies [8–10, 18–20]. However, our study design included only patients with HCV genotype 2.

A cutoff value of total cholesterol of 177 mg/dL in this study represented the best value in terms of sensitivity and specificity for SVR. Our cutoff total cholesterol level was lower than other previous studies [8–10, 18–20]. However, American Diabetes Association guidelines suggest that a goal should be a total cholesterol of <160 mg/dL in patient with type 2 diabetes who is at low risk [21]. Furthermore, Miller et al. reported that American type 2 diabetic patients had an average cholesterol level of 179 mg/dL [22].

The reason for SVR improvement in patients with elevated serum cholesterol levels is unknown. In patients with chronic hepatitis B and hepatitis C, serum lipid levels have been reported to be correlated with specific cytokines that may have antiviral activity, including tumor necrosis factor-alpha and interleukin-6 [23]. This hyperlipidemia-induced increase in cytokine levels may have a favorable and potentially additive effect on antiviral treatment in patients with chronic hepatitis C. Another proposed mechanism may be related to a possible regulatory effect of cholesterol in HCV binding to cell surface receptors, which in turn may be relevant to viral clearance [24]. The LDL receptor, a membrane glycoprotein, has been shown to be involved in HCV entry into hepatocytes, and data suggest that HCV RNA levels correlate with LDL receptor expression [25, 26]. Elevated serum concentrations of LDL may decrease the number of LDL receptors located on hepatocytes. 

Recent studies have shown that single nucleotide polymorphisms located in the gene region encoding interleukin 28b (IL28B) are strongly associated with the response to PEG-IFN and ribavirin therapy [17, 27, 28]. Total cholesterol, LDL cholesterol, and ApoB concentrations are significantly higher in chronic hepatitis C patients carrying a second IL28B major allele (CC in rs 12979860) compared with those possessing minor alleles (CT or TT) [29]. Therefore, the association between serum LDL cholesterol concentration and SVR may be reflected by the underlying link between IL28B genotypes and LDL cholesterol concentrations. As discussed above, we cannot exclude the possibility that high cholesterol levels in patients with HCV only reflect the presence of the IL28B major allele. It may simply reflect the wild-type sequence at core amino acid 70 because substitution in the core protein correlated significantly with a low concentration of LDL cholesterol [30, 31]. However, we could not identify IL28-B and core amino acid 70 as predictive for our patients with HCV genotype 2 because our sample population was limited. 

Petta and Craxì reported that age, sex, stage of fibrosis, and baseline viral load were important predictive factors SVR in patient with HCV genotype 2 [32]. However, our study was not significantly differente in these factors for SVR. Furthermore, there was no significant difference in the serum TC between SVR and non-SVR by the Mann-Whitney *U* test. The reason may be explained as follows: severe stage of fibrosis (F3-4) was recruited only 15%, and 25% was stage unknown in this study. HCV RNA in high TC group was significant higher than low TC group. Finally, this was the limitation small sample size and retrospective study. The discrepancies of the observation from different reports need further investigation.

 In conclusion, our data suggest that baseline serum total cholesterol levels should be considered when assessing the likelihood of sustained treatment response following PEG-IFN and ribavirin therapy in patients with chronic HCV genotype 2 infection. However, this finding requires further analysis.

## Figures and Tables

**Table 1:** Characteristics of 100 studied patients with HCV genotype 2.

All	100	
Age	57.0	(24–76)
Sex (%)		
Male	54	(54)
Female	46	(46)
Height (cm)	162	(138–186)
Weight (kg)	58	(37–87)
BMI (kg/m^2^)	22.7	(18.4–30.8)
Clinical finding (%)		
Chronic hepatitis	93	(93)
Cirrhosis	7	(7)
WBC (/*μ*L)	5100	(2100–9730)
Hemoglobin (g/dL)	14.0	(10–16)
Platelet (10^4^/*μ*L)	20.4	(6.9–26.5)
AST (IU/L)	42	(17–157)
ALT (IU/L)	52	(11–280)
TC (mg/dL)	177	(106–269)
<177 mg/dL (%)	50	(50)
≥177 mg/dL (%)	50	(50)
TG (mg/dL)	88	(56–262)
<88 mg/dL (%)	50	(50)
≥88mg/dL (%)	50	(50)
LDL-C (mg/dL)	98	(30–167)
<98 mg/dL (%)	50	(50)
≥98 mg/dL (%)	50	(50)
HCV RNA (KIU/mL)	1000	(20–40900)
Distribution of stage of fibrosis (%)		
0-1	43	(43)
2	17	(17)
3	11	(11)
4	4	(4)
Unknown	25	(25)
Distribution of grade of inflammation (%)		
0-1	39	(39)
2	34	(34)
3	2	(2)
Unknown	25	(25)
Treatment period (week) (%)		
<24	10	(10)
24	83	(83)
25–48	5	(5)
>48	2	(2)
Therapeutic efficacy (%)		
SVR	74	(74)
Non-SVR	26	(26)

Data are median (range) or frequency (%).

**Table 2:** Factors associated with response to peginterferon alfa-2b and ribavirin therapy.

	SVR	(Range or %)	Non-SVR	(Range or %)	*P* value
Total	74		26		
Age (y.o.)	57	(24–72)	57	(31–78)	NS
Sex (%)					
Male	33	(45)	13	(50)	
Female	41	(55)	13	(50)	NS
BMI (kg/m^2^)	23.1	(15.4–30.9)	21.0	(18.4–26.0)	NS
WBC (/*μ*L)	5100	(2100–9730)	5145	(3000–8300)	NS
Hemoglobin (g/dL)	14.1	(10–16)	14.0	(10–16)	NS
Platelet (10^4^/*μ*L)	21.7	(6.9–26.5)	11.5	(7.3–21.1)	NS
AST (IU/L)	39	(17–377)	44	(17–140)	NS
ALT (IU/L)	51	(11–751)	53	(14–169)	NS
TC (mg/dL)	183	(106–269)	163	(127–248)	NS
<177 mg/dL (%)	31	(42)	19	(73)	
≥177 mg/dL (%)	43	(58)	7	(27)	0.005
TG (mg/dL)	98	(56–262)	83	(74–176)	NS
<88 mg/dL (%)	33	(44)	17	(67)	
≥88 mg/dL (%)	41	(56)	9	(33)	NS
LDL-C (mg/dL)	109	(30–167)	88	(64–117)	0.015
<98 mg/dL (%)	30	(40)	20	(77)	
≥98 mg/dL (%)	44	(60)	6	(23)	0.020
HCV RNA (KIU/mL)	1000	(20–40900)	1850	(37–24200)	NS
Distribution of stage of fibrosis (%)					
1	31	(42)	12	(46)	
2	14	(19)	3	(12)	
3	6	(8)	5	(19)	
4	3	(4)	1	(4)	
Unknown	20	(27)	5	(19)	NS
Distribution of grade of inflammation (%)					
1	27	(36)	12	(46)	
2	25	(34)	9	(35)	
3	2	(3)	0	(0)	
Unknown	20	(27)	5	(19)	NS

Data are median (range) or frequency (%).

**Table 3:** Univariate and multivariate analysis of the factors associated with SVR to peginterferon alfa-2b and ribavirin therapy.

		Univariate analysis		Multivariate analysis	
		*P*	RR (95% CI)	*P*	RR (95% CI)
Age	<57 years	0.646	1.24 (0.50–3.09)		
Sex	Female	0.634	0.80 (0.33–1.97)		
BMI	≥23 kg/m^2^	0.221	1.86 (0.69–5.02)		
Underlying liver disease					
	CH	0.872	1.15 (0.21–6.32)		
WBC	≥5100 /*μ*L	0.827	0.75 (0.37–2.22)		
Hb	≥14.0 g/dL	0.317	0.62 (0.25–1.58)		
Plt	≥20 × 10^4^/*μ*L	0.112	2.10 (0.84–5.24)		
AST	<40 IU/L	0.429	1.44 (0.58–3.55)		
ALT	<52 IU/L	0.649	1.23 (0.50–3.02)		
*γ*-GTP	<35 IU/L	0.525	0.75 (0.30–1.83)		
TC	≥177 mg/dL	0.008	3.77 (1.41–10.05)	0.015	18.59 (1.78–193.65)
TG	≥88 mg/dL	0.101	2.60 (0.83–8.13)		
LDL-C	≥98 mg/dL	0.028	4.91 (1.19–20.23)	0.800	1.25 (0.22–7.01)
Stage	F 3-4	0.419	0.60 (0.17–2.07)		
Grade	A 2-3	0.809	1.13 (0.41–3.18)		
HCV RNA					
	<1000 KIU/mL	0.310	1.65 (0.63–4.31)		

Relative risk (RR); 95% confidence interval (95% CI).

**Table 4:** Comparison between HCV patients with high and low serum TC.

TC	<177 mg/dL	(Range or %)	≥177 mg/dL	(Range or %)	*P* value
Total	50		50		
Age (y.o.)	57	(24–78)	57	(36–69)	NS
Sex (%)					
Male	34	(68)	20	(40)	
Female	16	(32)	30	(60)	0.005
BMI (kg/m^2^)	21.5	(18.4–26.8)	23.5	(15.4–30.6)	0.027
WBC (/*μ*L)	5100	(2100–9730)	5100	(3000–8300)	NS
Hemoglobin (g/dL)	14.2	(10–16)	13.9	(10–16)	NS
Platelet (10^4^/*μ*L)	17.6	(7.3–26.5)	21.7	(6.9–26.1)	NS
AST (IU/L)	48	(17–377)	33	(18–199)	0.033
ALT (IU/L)	67	(16–751)	40	(11–283)	NS
TG (mg/dL)	83	(46–203)	111	(43–262)	NS
<88 mg/dL (%)	32	(63)	19	(38)	
≥88 mg/dL (%)	18	(37)	31	(62)	0.045
LDL-C (mg/dL)	84	(43–118)	121	(30–167)	<0.001
<98 mg/dL (%)	40	(79)	9	(19)	
≥98 mg/dL (%)	10	(21)	41	(81)	<0.001
HCV RNA (KIU/mL)	1000	(20–24200)	2670	(20–40900)	0.029
Distribution of stage of fibrosis (%)					
1	18	(36)	25	(50)	
2	7	(14)	10	(20)	
3	8	(16)	3	(6)	
4	2	(4)	2	(4)	
Unknown	15	(30)	10	(20)	NS
Distribution of grade of inflammation (%)					
1	20	(40)	19	(38)	
2	14	(28)	20	(40)	
3	1	(2)	1	(2)	
Unknown	15	(30)	10	(20)	NS

Data are median (range) or frequency (%).
